# In vitro inhibition of human influenza A virus replication by chloroquine

**DOI:** 10.1186/1743-422X-3-39

**Published:** 2006-05-29

**Authors:** Eng Eong Ooi, Janet Seok Wei Chew, Jin Phang Loh, Robert CS Chua

**Affiliations:** 1 Defence Medical and Environmental Research Institute, DSO National Laboratories, 27 Medical Drive, #09-01, 117510, Singapore

## Abstract

Chloroquine is a 9-aminoquinolone with well-known anti-malarial effects. It has biochemical properties that could be applied to inhibit viral replication. We report here that chloroquine is able to inhibit influenza A virus replication, in vitro, and the IC50s of chloroquine against influenza A viruses H1N1 and H3N2 are lower than the plasma concentrations reached during treatment of acute malaria. The potential of chloroquine to be added to the limited range of anti-influenza drugs should be explored further, particularly since antiviral drugs play a vital role in influenza pandemic preparedness.

## Findings

Antiviral drugs against influenza virus play an important role in the treatment and prevention of human influenza infection. The adamantanes have been used for decades and resistance to this class of drugs has become prevalent in some parts of the world [1]. The neuraminidase inhibitor, oseltamivir, is currently regarded as the first line of defence against a pandemic until a suitable vaccine can be produced in sufficient quantities. Emergence of resistance to this drug in human influenza A viruses [2], as well as the H5N1 subtype in Vietnam [3] is thus a cause for concern. Resistance has not been reported for zanamivir, another neuraminidase inhibitor [4]. Expanding the range of antiviral drugs that effectively inhibit influenza A virus replication is thus a matter of urgency.

A recent review has suggested that the anti-malarial drug, chloroquine, may have antiviral activity [5]. As a lysosomotropic weak base, it impairs replication of some viruses through reducing the efficiency of endosome-mediated virus entry or through inhibiting the low-pH dependent proteases in trans-Golgi vesicles [5]. Its antiviral activities against the human immunodeficiency virus (HIV) [6] and the SARS coronavirus have been demonstrated [7,8]. Previously, chloroquine had been used to study influenza virus replication in vitro [9]. However, the 0.1 mM concentration used was too high to indicate its therapeutic usefulness [9]. We thus carried out an in vitro antiviral assay to determine the 50% and 90% inhibitory concentration (IC50 and IC90, respectively) of chloroquine against influenza A virus subtypes H1N1 and H3N2.

The in vitro antiviral screening assay was modified from a previously described method [10] and carried out in triplicates. Influenza A viruses H1N1 (ATCC: VR1520) and H3N2 (ATCC: VR544) were used in this study. Briefly, 50 μl of serial 2-fold dilutions of the chloroquine were incubated overnight with 100 μl of MDCK cells giving a final cell count of 30,000 cells per well in a 96-well microtitre plate (Nunc A/S Roskilde, Denmark), for the drug to equilibrate with the cells. 50 μl of virus at a concentration of 100 50% tissue culture infectivity dose (TCID50) was added to each well and the plate incubated at 37°C in 5% CO_2 _for 2 days. Viral replication was assessed by 2 methods: real-time RT-PCR using a previously described protocol [11] was carried out on nucleic acid extracted from the supernatant of the culture, using the Roche Lightcycler system (Roche Diagnostics, Mannheim, Germany); FITC-labeled anti-nucleoprotein monoclonal antibody (Chemicon International, Temecula, CA) was used to stain the remaining cell monolayer and viewed under an ultraviolet light microscope. Controls consisting of virus and cell only, cell only, virus only and serial 2-fold dilution of amantadine in place of chloroquine were included in each plate. Cytotoxicity of the drugs was also assessed at all concentrations used in the antiviral assay.

The results are shown in Table 1 (see additional file 1). Cellular toxicity was observed at a chloroquine concentration of 25 μM but not at higher dilutions. Complete inhibition of influenza A virus H1N1 and H3N2 replication was observed at 12.5 μM chloroquine. This was evident microscopically with the absence of immunofluorescent focus as well as a similar Ct value on real-time RT-PCR as the virus only control. The number of fluorescent foci increased with decreasing concentration of chloroquine and this is similarly reflected in the decrease in Ct value of the real time RT-PCR of their respective supernatant. The IC50 and IC90 of chloroquine against H1N1 are 3.6 μM and 9.9 μM, respectively, while those for H3N2 are 0.84 μM and 2.4 μM, respectively.

Only a handful of drugs are able to inhibit influenza A virus replication and the increasing prevalence of resistance to these drugs demands newer classes of anti-influenza drugs. Although new compounds are still being developed and their anti-influenza activity assessed, these may take years to fulfill the regulatory requirements before clinical use can be initiated. An alternative to this mode of drug discovery may be to find new uses for old drugs, where the availability of extensive experience with their clinical use may shorten the duration needed for the various phases of clinical trials. Chloroquine serves as such an example. Our results suggest that chloroquine has a clinically useful inhibitory activity against influenza A virus replication.

The IC50 and IC90 for both the H1N1 and H3N2 viruses are lower than the plasma concentration of chloroquine that can be attained with dosages used in the prophylaxis and treatment of acute malaria [12]. This suggests that the dosages needed to inhibit human influenza A virus replication should be well tolerated by patients.

An added effect of chloroquine is its immunomodulatory activity [5]. This may have an added benefit for the treatment of influenza A (H5N1) infection since the pathology of avian influenza infection in humans appears to be mediated by pro-inflammatory cytokines [13].

Although we have shown that chloroquine is able to inhibit two reference subtypes of influenza A viruses, testing the susceptibility of a wide range of clinical isolates of influenza A viruses to chloroquine, as well as in vivo studies, would be needed to determine the role, if any, of chloroquine in the prophylaxis and treatment against influenza infection. It would also be interesting to determine the activity of chloroquine against the highly pathogenic H5N1 subtype as this would add to the very limited antiviral stockpile against a possible pandemic.

In conclusion, chloroquine demonstrates an inhibitory effect against the replication of human influenza A virus H1N1 and H3N2, in vitro and further studies to explore its therapeutic and prophylactic potential against influenza epidemics and pandemics should be encouraged.

## Abbreviations

TCID50: 50% tissue culture infectivity dose; IC50: concentration of chloroquine needed to inhibit 50% of viral growth; IC90: concentration of chloroquine needed to inhibit 90% of viral growth; RT-PCR: Reverse-transcription polymerase chain reaction. IFA: immunofluorescence assay.

## Competing interests

The author(s) declare that they have no competing interests.

## Authors' contributions

EEO designed the study and wrote the manuscript. JSWC carried out the real-time RT-PCR. JPL set up and validated the real-time RT-PCR for influenza in our laboratory. RCSC carried out the virus culture, antiviral assay and IFA.
